# Toward gender equity in contraception: male contraceptive passes safety trial

**DOI:** 10.1016/j.ebiom.2025.105938

**Published:** 2025-09-10

**Authors:** eBioMedicine

Contraceptives have existed, in some form or another, throughout human history, driven by a fundamental need to support individual reproductive choice and manage resources. Contraceptives have ranged from the documented use of crude versions in Ancient Egypt—such as honey, acacia leaves, and lint to block sperm—to the female oral combined contraceptive pill, which underwent trials in the 1950s and was approved as the first pharmaceutical contraceptive in 1960.

Contraceptives have been geared primarily towards women, with options for men limited to barrier methods such as condoms and surgical interventions, such as vasectomy, which is not always reversible. Female contraceptive options include oral contraceptives, subdermal implants, intrauterine devices and systems, and barrier methods such as diaphragms. Many of these contraceptive options are hormonal interventions, and have numerous potential side effects of varying degrees on female bodies. When the first oral contraceptive, Enovid, was approved in 1960, it was found that 25% of all women who used it discontinued use due to side effects—including nausea, headaches, bloating, weight gain, and, most concerningly, thromboembolisms. Such side effects have been reduced with the recommended lower doses in use today, but still remain. There is the potential osteoporosis associated with long-term use of depot medroxyprogesterone acetate, another hormonal contraceptive method, and intrauterine devices can increase the risk of STIs and painful, heavy periods. Finally, there is the heavy burden of pregnancy and childbirth and their associated risks. However, the health and social benefits continue to outweigh the risks for most women, including decreased risk of various types of cancer including benign breast, endometrial, and ovarian cancer; plus, moderating excessive menstrual and anovulatory bleeding, which occurs those with PCOS, and provides relief for dysmenorrhea and pain symptoms associated with endometriosis.

A continued push for unrestricted access to contraceptives for all is needed. Part of this effort should focus on the development of expanding effective contraceptive options for the other half of the population; namely, men. There have been numerous attempts and trials, none of which have yet materialised into an approved male contraceptive. Various hormonal interventions have been examined; testosterone enanthate underwent two major multicentre clinical trials in the 1980s, one in 271 couples published by *The Lancet,* and one in 399 couples published in *Fertility and Sterility**,* aiming to establish the suppression of spermatogenesis and identify whether it would prevent pregnancies. It was shown to be highly effective, resulting in only 0·8 pregnancies per 100 person-years of exposure. However, it never materialised into an approved contraceptive option, partly due to the method of delivery (weekly intramuscular injections) and the long duration (months, sometimes up to a year) required before sperm concentrations were adequately suppressed. A large, multicentre efficacy trial was done to examine the combination of norethindrone enanthate and testosterone undecanoate, a promising combined testosterone and progestin treatment that had resulted in suppression to less than 1 million/mL sperm (ie, the level clinically classified as severe oligozoospermia) in more than 90% of men for up to 48 weeks. The phase 2B trial showed that this contraceptive was able to efficiently suppress sperm—to less than or equal to 1 million/mL in 274 of 320 men, and only four pregnancies occurred. However, the trial was stopped prematurely for safety reasons, including mood changes and depression, both of which are side effects of existing female oral contraceptives, especially older ones containing ethinylestradiol.

Non-hormonal male contraceptives have also been investigated since the 1960s. Several options have been explored, including gossypol, a polyphenol isolated from the cotton plant, which can curb spermatogenesis, is well tolerated, and has no notable short-term side-effects, but unfortunately resulted in spermatogenesis not fully reversing in 50% of men. WIN 18,446, an inhibitor of ALDH1A1/1A2 enzymes that synthesise retinoic acid, which regulates male germ cell development and differentiation and thereby blocking spermatogenesis, resulted in a number of side-effects when combined with alcohol, including nausea, vomiting, palpitations, and sweating, and thus both were abandoned.

New promise is shown, however, in a non-hormonal male contraceptive, YCT-529. As a retinoic acid receptor-α antagonist, it functions by impairing retinoic acid signalling in the testes. As such, it decreases sperm count and may be able to prevent pregnancy. It had already been shown to disrupt spermatogenesis in mice within 4 weeks of oral administration, resulting in temporary infertility, and a full reversal within 6 weeks of cessation.

As a consequence of the findings in mice, a safety trial in humans was initiated. YCT-529 was shown, in a small phase 1A trial published in *Communications Medicine**,* to have no effect on heart rate, hormones, sex-hormone binding globulin, inflammatory biomarker levels, sexual desire, or mood, in 16 male vasectomised volunteers who received 10, 30, 90, or 180 mg single oral doses of the contraceptive. However, further studies are required to ensure its effectiveness in preventing pregnancy, as well as to understand the mechanisms of action behind such contraceptives, such as one study in a mouse model attempting to further characterise the interactions between retinoic acid receptors and silencing mediator of retinoid and thyroid hormone receptors in spermatogenesis.

Differences in the tolerance and acceptability of side effects between male and female contraceptives highlight ongoing gender disparities in health research priorities.

And yet, these recent findings are a step in the right direction; both men and women want and would accept male contraceptives; women trust their partners to use it, and men want autonomy over their family planning, shown by the 44–83% of men across different countries that said they would use a contraceptive pill. The ability of men to have autonomy over their own reproductive planning is important, and would reduce the burden on women, and—at a time when access to contraceptives is being threatened, when they are needed by so many—would help to move us further towards the goal of unrestricted access to contraceptives for all. *eBioMedicine* encourages the submission of research that aims to characterise the efficacy and safety of all contraceptives, as well as understand the mechanisms behind their method of action.

